# NFAT5: A Metabolic Time Capsule Encoding the History of Paternal Metabolic Oxidative Stress Within the Male Reproductive Tract

**DOI:** 10.3390/antiox15050645

**Published:** 2026-05-20

**Authors:** Nicola Mosca, Antonella Migliaccio, Teresa Chioccarelli, Donato Cappetta, Antonella De Angelis, Marialucia Telesca, Liberato Berrino, Danila Valletta, Alice Luddi, Chiara Donati, Paola Piomboni, Charles Coutton, Guillaume Martinez, Gilda Cobellis, Chiara Schiraldi, Nicoletta Potenza, Rosanna Chianese, Francesco Manfrevola

**Affiliations:** 1 Department of Environmental, Biological, Pharmaceutical Sciences and Technologies, University of Campania “Luigi Vanvitelli”, 81100 Caserta, Italy; nicola.mosca@unicampania.it (N.M.); danila.valletta@unicampania.it (D.V.); 2 Department of Experimental Medicine, University of Campania “Luigi Vanvitelli”, 80138 Naples, Italy; antonella.migliaccio@unicampania.it (A.M.); teresa.chioccarelli@unicampania.it (T.C.); antonella.deangelis@unicampania.it (A.D.A.); marialucia.telesca@unicampania.it (M.T.); liberato.berrino@unicampania.it (L.B.); gilda.cobellis@unicampania.it (G.C.); chiara.schiraldi@unicampania.it (C.S.); francesco.manfrevola@unicampania.it (F.M.); 3 Department of Experimental Medicine, University of Salento, 73100 Lecce, Italy; donato.cappetta@unisalento.it; 4 Department of Molecular Medicine and Development, University of Siena, 53100 Siena, Italy; alice.luddi@unisi.it (A.L.); paola.piomboni@unisi.it (P.P.); 5 Department of Experimental and Clinical Biomedical Sciences “Mario Serio”, University of Florence, 50100 Florence, Italy; chiara.donati@unifi.it; 6 Hôpital Couple-Enfant, Centre Hospitalier Universitaire de Grenoble, UM de Génétique Chromosomique, 38000 Grenoble, France; ccoutton@chu-grenoble.fr (C.C.); gmartinez@chu-grenoble.fr (G.M.); 7 Genetic Epigenetic and Therapies of Infertility, Institute for Advanced Biosciences INSERM U1209, CNRS UMR5309, 38000 Grenoble, France

**Keywords:** circRNAs, NFAT5, male infertility, HFD-oxidative stress, intergenerational inheritance

## Abstract

Leydig cells (LCs) represent a somatic testicular population responsible for testosterone synthesis, a hormone essential for spermatogenesis and male fertility. The obesity condition impairs LC steroidogenic activity, contributing to testicular oxidative stress and male reproductive dysfunctions. Using a high-fat-diet (HFD) murine model, we investigated the regulatory role of the nuclear factor of activated T cells 5 (NFAT5s) in the obesity-induced LC damage and the resulting alterations in intergenerationally inherited sperm circRNA cargo. Our findings reveal a significant upregulation of both *circNFAT5* and NFAT5 protein levels in HFD testis. This molecular signature correlated with decreased antioxidant defense system, increased LC apoptosis, and impaired steroidogenesis. In vitro experiments, performed in TM3 cells, confirmed that NFAT5 nuclear shuttling drives proapoptotic gene activation, while *NFAT5* silencing promotes LC survival. The analysis of HFD progeny (F1H) revealed a full recovery of testis oxidative status and LC apoptosis, linked with the recovery of NFAT5 expression. However, a steroidogenic deficiency persisted in F1H offspring. Notably, HFD and F1H epididymides exhibited NFAT5 overexpression concomitantly with impaired sperm morphology, motility, viability, and altered sperm circRNA profiles alongside a deregulated 4-hydroxy-2-nonenal (4HNE) profile, a marker of sperm oxidative stress. Lastly, an enhanced FUS-related amplification of circRNA perturbations was highlighted in F1H spermatozoa. Collectively, our findings reveal a dual functional role of NFAT5 as a testicular regulator of LC fate and an epididymal sentinel of metabolic stress, in turn linking paternal obesity to the persistent transmission of sperm epigenetic anomalies across the offspring.

## 1. Introduction

As the predominant steroidogenic population in the testicular interstitium, Leydig cells (LCs) are responsible for the majority of androgen production, the hormone essential for proper spermatogenesis [1,2]. Testosterone acts *via* androgen receptors (ARs) modulating multiple germ cell activities, including: (i) the maintenance of blood–testis barrier (BTB) integrity, (ii) the meiotic progression of germ cells, (iii) the adhesion of spermatids to Sertoli cells (SCs), and (iv) the sperm release [2,3,4]. Consequently, any pathophysiological condition impairing LC physiology or steroidogenic function could converge in reproductive defects.

Obesity exerts significant detrimental effects on LC steroidogenic capacity. Obese men frequently exhibit low sperm count, reduced sperm quality, and sexual dysfunctions [5,6,7,8]. Such reproductive anomalies are thought, at least in part, to arise from disrupted testicular steroidogenesis, as obesity-associated low testosterone levels closely correlate with reproductive disorders, including a high prevalence of secondary (hypogonadotropic) hypogonadism [9,10,11,12].

Mounting evidence underscores how obesity orchestrates a decline in LC steroidogenic integrity. This dysfunction is fundamentally driven by the confluence of oxidative disequilibrium, inflammatory infiltration, and the induction of programmed cell death within the specialized testicular microenvironment [13,14,15]. Accordingly, significant impairments in both expression and activity of major antioxidant enzymes, including catalase (CAT), superoxide dismutase (SOD), and glutathione peroxidase (GPX), have been consistently reported in multiple experimental models of diet-induced obesity [13,14,15], highlighting a close functional link between obesity and the dysregulation of the testicular antioxidant defense system.

However, although several studies have effectively documented obesity-related abnormalities in LC structure, mitochondrial integrity, and oxidative status [13,16,17], a deep mechanistic framework explaining how obesity disrupts LC homeostasis remains elusive. Hence, the identification of molecular biosensors constitutes a critical endeavor in discerning early functional derangements in the LC population.

Alongside all that, spermatozoa (SPZ) serve as a great reservoir of epigenetic information through a circular RNA (circRNA) cargo, recently emerged as promising candidate capable of capturing and reflecting the upstream testicular perturbations [18]. Interestingly, in our previous work, we have characterized the sperm circRNA landscape in a high-fat-diet (HFD)-induced obesity murine experimental model and identified a panel of differentially expressed (DE-) circRNAs implicated in the regulation of sperm function and quality [19]. Among the identified DE-circRNAs in HFD SPZ, *circNFAT5* has emerged as a molecule of particular interest with striking expression divergence—upregulated in the testis and downregulated in SPZ—thus suggesting a potential role at the interface between testicular dysfunction and sperm quality [19].

The family of nuclear transcription factors of activated T cells (NFAT) comprises five members (NFAT1–NFAT5), all sharing a conserved DNA-binding domain and regulating several biological programs [20]. Among them, NFAT2 has been identified as an isoform expressed in LCs, where its activation promotes FasL expression and drives apoptosis activation [21]. Building on this context, the present study aimed to investigate whether the sperm *circNFAT5* epigenetic signature could serve as a readout of oxidative stress-driven LC dysfunctions, potentially reflecting upstream molecular pathways orchestrated by the canonical protein isoform NFAT5.

To achieve our objective, we leveraged a high-fat-diet (HFD) murine model to comprehensively characterize circNFAT5 and its protein counterpart, NFAT5, in the testis. This approach aimed to define the NFAT5-dependent molecular pathways that potentially exacerbate LC apoptosis under metabolic stress. Furthermore, by extending our analysis to the epididymal compartment, we explored the role of NFAT5 in post-testicular sperm maturation. This allowed us to assess both morpho-functional and epigenetic changes, providing a clearer definition of how obesity-induced conditions remodel the sperm circRNA cargo.

Our findings uncover an unrecognized mechanistic pathway underlying the obesity-induced LC damage, while highlighting the potential of sperm *circNFAT5* as an accessible bivalent sentinel marker for simultaneously evaluating testicular endocrine functions and sperm epigenetic quality.

## 2. Materials and Methods

### 2.1. Experimental Animals

This study was performed by using eight-week-old Mus musculus C57BL6/J mice (ENVIGO Srl, Udine, Italy). The mice were randomly assigned to receive a normal-fat diet (CTRL, n = 12; Teklad Standard TD.2918; carbohydrate, 58%; protein, 24%; fat, 18% Kcals; ENVIGO) or high-fat diet (HFD, n = 12; Teklad Custom Research Diet TD. 06414; carbohydrate, 21%; protein, 18%; fat, 60% Kcals; ENVIGO) for 12 weeks, according to previously validated experimental strategy [19]. At the end of dietary regimen, six males from each group (n = 6) were mated 1:1 with young female mice to obtain F1 generation. Pregnant females were maintained on a control diet until the second week of lactation. To rigorously account for potential litter effects, two male F1 offspring were randomly selected from each of the six independent litters per group (n = 12 F1-CTRL and n = 12 F1-HFD, the latter referred to as F1H).

After weaning, the F1 offspring were given free access to control diet and water. Three primary cohorts were established for downstream analyses: CTRL (F0), HFD (F0), and F1H offspring. F1 offspring from the CTRL group were excluded from further investigation, as the study aimed specifically to characterize the paternal transmission of HFD-induced effects relative to the paternal baseline. Given the complexity of the experimental design, animals were allocated into subgroups to ensure that each morphological and molecular analysis was performed on n = 6 independent biological replicates for each experimental group (CTRL, HFD, and F1H). This rigorous distribution was essential to guarantee both statistical significance and the highest degree of robustness for each investigated parameter. The animals were sacrificed by cervical dislocation when completely sedated with 4% isoflurane (Iso-Vet, Piramal Healthcare UK Limited, Morpeth, UK) for 5 min in a Plexiglass chamber, after making sure of the lack of heartbeat and reflex active paw. The testes were rapidly removed and stored at −80 °C for molecular investigations and/or fixed in Bouin’s solution for morphological analyses. The epididymides were removed and processed for the collection of *cauda* SPZ for molecular investigations and/or fixed in Bouin’s solution for morphological analyses.

### 2.2. Ethical Approval

All procedures involving animal care were performed in accordance with the National Research Council’s publication *Guide for Care and Use of Laboratory Animals* (National Institutes of Health Guide). The Italian Ministry of Education and the Italian Ministry of Health approved the experiments with the authorization no. 405/2021-PR (7 June 2021).

### 2.3. Sperm Collection

*Cauda* epididymis collected from CTRL, HFD, and F1H mice (n = 6 animals for each experimental condition) were processed as previously described [19]. The epididymis was cut in PBS (pH 7.6) to let SPZ flow out from the ducts. Samples were centrifuged at 1500× *g* for 30 min at 4 °C, and SPZ pellets were incubated on ice with somatic cell lysis buffer (SCLB; 0.1% SDS, 0.5% Triton X-100 in DEPC-H_2_O) to eliminate somatic cell contaminations. Following washes in PBS, aliquots of *cauda* SPZ were stored at −80 °C for molecular analyses or dried on slides for morphological investigations. For sperm functional analysis, aliquots of *cauda* SPZ were processed as reported above.

### 2.4. Sperm Morpho-Functional Analysis

SPZ collected from *cauda* epididymis of CTRL, HFD, and F1H mice (n = 6 animals for each experimental condition) were dried on slides and used for hematoxylin and eosin (H&E) staining according to standard procedures. Morphological analyses were performed under a light microscope (Leica CTR500, Leica Microsystems Inc., Milan, Italy) capturing images by using a high-resolution digital camera (Leica DC300F). Sperm morphological assessment was based on sperm head and tail anomalies, including: misshapen heads (loss or alteration of the hook structure), tapered or pyriform heads, defective head–neck alignment, and bent or coiled tails.

The number of motile and non-viable SPZ was investigated using a hemocytometer (Burker Chamber). For the total SPZ number, 20 fields per sample were analyzed. The viable dye Trypan blue reagent (Trypan Blue, 0.4% Solution, 17-942E Lonza) was used to evaluate the number of non-viable SPZ, expressed as percentage of non-viable/total SPZ (mean value ± SEM). Sperm viability criteria were based on membrane integrity and permeability to Trypan blue reagent; this leads to classifying unstained SPZ as viable cells and stained SPZ as non-viable cells.

Motile SPZ were reported as a percentage of motile/live SPZ (mean value ± SEM). A minimum of 150 sperm cells was counted for each analysis (n = 6 animals for each experimental condition). Sperm motility was evaluated by direct observation under light microscopy and classified as motile or non-motile based on the presence or absence of any flagellar movement within the observation field. All the results were validated twice by the same operator.

### 2.5. Sperm Acrosome and Oxidative Stress Analysis

SPZ collected from *cauda* epididymis of CTRL, HFD, and F1H mice (n = 6 animals for each experimental condition) were dried on slides and fixed in 4% paraformaldehyde (sc-281692; Santa Cruz Biotechnology, Heidelberg, Germany) for 20 min at ambient temperature (RT). For acrosome analysis, samples were permeabilized with 0.1% Triton X-100 (X100; Sigma-Aldrich, Milano, Italy) and incubated with peanut agglutinin (PNA) lectin (L32458; Alexa Fluor 568, Thermo Fisher Scientific, Waltham, MA, USA), diluted 1:50, for 1 h at 37 °C. For sperm oxidative stress assessment, samples were permeabilized with 0.1% Triton X-100, and a blocking step was conducted with 10% donkey serum (ab7475; Abcam, Cambridge, UK) for 30 min at RT. Slides were then incubated with anti-4HNE antibody (ab46545; Abcam, USA) overnight (ON) at 4 °C. Following three washes in PBS, a conjugated secondary antibody was used (Jackson ImmunoResearch, Cambridge, UK) for 1 h at 37 °C. Nuclei were labeled with DAPI (D9542; Sigma-Aldrich, Milano, Italy), and the slides were analyzed under an optical microscope (Leica DM 5000 B + CTR 5000; Leica Microsystems, Wetzlar, Germany) with a UV lamp as reported above. Densitometric analysis of 4HNE immunofluorescence was performed with ImageJ Software (version 1.53 g) and adjusted relative to DAPI fluorescence intensity. A minimum of 150 sperm cells was counted for each assay to determine the amount of PNA positive cells and the quantification of 4HNE signal. Data were reported as the percentage of PNA positive cells and 4HNE fluorescence intensity (mean value ± SEM). All the results were validated twice by the same operator.

### 2.6. Steroid Hormones Intratesticular Dosage

The intratesticular testosterone (TT) and 17-β-estradiol (E_2_) dosage were performed in CTRL, HFD, and F1H mice (n = 6 animals for each experimental condition) by LC-MS analysis according to previously validated protocols [22]. In brief, a liquid–liquid extraction coupled to a solid-phase extraction on AFFINIMIP^®^ SPE ESTROGENS cartridges (Polyntell SA, Paris, France) was carried out. Then, a Dionex UltiMate 3000 HPLC system (Thermo Fisher Scientific Inc, Rodano, Italy) coupled to a triple quadrupole mass spectrometer (API 2000, AB Sciex, Darmstadt, Germany) was used for sample analysis. A Kinetex F5 (100 × 4.6 mm, 2.6 μm) stainless steel column (Phenomenex, Bologna, Italy) was used for reversed-phase separations. The analytes were quantified in multiple reaction monitoring mode. Data were reported as hormone ng/g tissue (mean value ± SEM).

### 2.7. Cell Culture, Transfections, and Treatment

Murine Leydig cell line TM3 (Icellbioscience iCell-m058) was cultured in DMEM/F12 (1:1) supplemented with 2.5% fetal bovine serum, 5% horse serum, 100 U/mL penicillin, and 100 μg/mL streptomycin. Cultures were routinely tested monthly to confirm the absence of mycoplasma contamination.

For transfection, cells were seeded the day before in antibiotic-free medium and transfected at 80–90% confluence with different doses (25 nM, 50 nM, 100 nM) of Mission^®^ esiRNA NFAT5 (EMU051441, Merck, Darmstadt, Germany) or Mission^®^ siRNA Universal Negative Control #1 (SIC001, Merck) (n = 3 for each experimental condition) using 2 µL of Lipofectamine 2000 (Invitrogen, Thermo Fisher Scientific, Waltham, MA, USA) per 1 µg of nucleic acids. Six h post-transfection, the transfection mix was replaced with complete growth medium. Analyses were performed 48 h after transfection. To induce apoptosis, TM3 cells were exposed to increasing doses of H_2_O_2_ (0–400 μM) according to previously validated protocols [23,24,25] and incubated for 4, 8, and 24 h.

### 2.8. Immunohistochemistry Analysis

Testis and *cauda* epididymis collected from (i) CTRL, HFD, and F1H mice (n = 6 animals for each experimental condition) were fixed ON in Bouin’s solution and embedded in paraffin according to standard procedure. For immunohistochemistry staining, tissue sections (7 μm thick) were deparaffinized, rehydrated, and permeabilized with PBS pH 7.4 containing 0.1% Triton X-100. Citrate buffer in the amount of 0.01 M (pH 6.0) was used for antigen retrieval, and a blocking step was performed using a PBS blocking solution containing 5% BSA and normal goat serum (diluted 1:5). The sections were incubated ON at 4 °C with anti-NFAT5 antibody (sc-398171, Santa Cruz Biotechnology, Heidelberg, Germany) diluted 1:100. The avidin/biotin and the substrate/chromogen H_2_O_2_/DAB system were used to reveal the immunoreactivity. Slides were observed under a light microscope (Leica CTR500, Leica Microsystems Inc., Milan, Italy). The images were captured using a high-resolution digital camera (Leica DC300F).

### 2.9. TUNEL Assay

Testis collected from (i) CTRL, HFD, and F1H mice (n = 6 animals for each experimental condition) were fixed and embedded in paraffin according to standard procedure. Testis sections (5 µm thick) were deparaffinized, rehydrated, and fixed in 4% paraformaldehyde at 4 °C for 1 h and rinsed in PBS pH 7.4. Apoptotic cells were detected using an in situ apoptosis detection kit (fluorescein; Takara Bio Inc., Kusatsu, Shiga, Japan) according to the manufacturer’s instructions. This assay is based on the labeling of DNA strand breaks with fluorescein-dUTP, which is catalyzed by terminal deoxynucleotidyl transferase (TdT). The sections were treated with proteinase K (20 µg/mL) for 15 min at RT. Then, they were rinsed in PBS and incubated with the equilibration buffer for 10 min. Finally, the sections were incubated with the TdT reaction mixture for 1 h at 37 °C. Negative controls were processed without TdT. After stopping the reaction with the stop solution, the nuclei were labeled with DAPI and mounted with Vectashield antifade medium. TUNEL-positive nuclei were analyzed using a confocal microscope (LSM700; Zeiss, Jena, Germany).

### 2.10. Protein Extraction and Western Blot Analysis

Total protein lysates from (i) CTRL, HFD, and F1H testes; (ii) CTRL, HFD, and F1H *cauda* epididymis; (iii) CTRL, HFD, and F1H *cauda* SPZ (n = 6 animals for each experimental condition); and iv) TM3 cells *in vitro* treated with a siRNA against NFAT5 or with H_2_O_2_ (n = 3 for each experimental condition) were obtained by using RIPA extraction buffer as previously reported [19]. Following protein extraction, the protein amount was assessed using the Lowry assay. An equal concentration was separated by SDS-PAGE and transferred to polyvinylidene difluoride membrane (GE Healthcare, Milano, Italy) at 280 mA for 2.5 h at 4 °C. The blocking step (5% nonfat milk, 0.25% Tween 20 in Tris-buffered saline) was carried out for 3 h at RT. Then, the filters were incubated with different primary antibodies (anti-FUS PA5-52610, Invitrogen, Milano, Italy; anti-TUBULIN ab15246, Abcam, Cambridge, UK; anti-P53 ab238069, Abcam, Cambridge, UK; anti-NFAT5 sc-398171, Santa Cruz Biotechnology, Heidelberg, Germany; anti-H3 ab10799, Abcam, Cambridge, UK; anti-BAX ab216494, Abcam, Cambridge, UK; anti-BCL2 sc-7382, Santa Cruz Biotechnology, Heidelberg, Germany; anti-CAT E-AB-11036, Elabscience; anti-SOD2 D-AB-10436L, Elabscience; anti-GPX1 E-AB-70149, Elabscience; anti-phosphoNFAT5 BS-9474R, Bioss, MA, USA; anti-STAR PA5-21687, Invitrogen, Milano, Italy; anti-LHR PA576197, Invitrogen, Milano, Italy; anti-HSD3β sc-515120, Santa Cruz Biotechnology, Heidelberg, Germany; anti-CYP19A1 E-AB-68290, Elabscience; anti-phosphoP53 44-640G Invitrogen, Milano, Italy) ON at 4 °C. After washing in 0.25% Tween 20/TBS, filters were incubated with 1:1000 horseradish peroxidase-conjugated mouse IgG (Dako Corp., Milano, Italy). An the enhanced chemiluminescence Western blotting detection system (Amersham ECL Western Blotting Detection Reagent (RPN2106), GE Healthcare, Milano, Italy) was used to detect the immune complexes. The signals were quantified by densitometry analysis using ImageJ software, adjusted relative to TUBULIN, and graphed in terms of optical density (OD) values as fold changes (mean ± SEM).

### 2.11. Total RNA Preparation

Total RNA was extracted from (i) CTRL, HFD, and F1H testes; (ii) CTRL, HFD, and F1H *cauda* SPZ (n = 6 animals for each experimental condition); and (iii) TM3 cells in vitro treated with a siRNA against NFAT5 or with H_2_O_2_ (n = 3 for each experimental condition) as previously described [19]. In brief, samples were homogenized using TRIzol Reagent (Invitrogen Life Technologies, Paisley, UK) following the manufacturer’s instructions. Then the homogenization, samples were mixed with chloroform and centrifuged at 12,000× *g* for 15 min at 4 °C. Total RNA was precipitated with isopropyl alcohol (0.5 mL per mL TRIzol) and 1 µL of glycogen (20 mg/mL). RNA pellets were washed with 75% ethanol, centrifuged at 7500× *g* for 10 min at 4 °C, and resuspended in DEPC-treated water for quantification and purity assessment (260/280 and 260/230 ratios) using a NanoDrop 2000 spectrophotometer (Thermo Fisher Scientific). RNA aliquots (10 µg) were treated with 2 U RNase-free DNase I to remove genomic DNA and stored at −80 °C.

### 2.12. RNA Expression Analysis by One-Step Quantitative RT-PCR

RNA expression analysis was performed in (i) CTRL, HFD, and F1H testes; (ii) CTRL, HFD, and F1H *cauda* SPZ (n = 6 animals for each experimental condition); and (iii) TM3 cells in vitro treated with H_2_O_2_ (n = 3 for each experimental condition) using a One-Step Evagreen RT-qPCR kit, which includes an RT-qPCR enzyme mix and Evagreen Mastermix (Applied Biological Materials Inc., Ferndale, WA, USA), on a CFX-96 Real-Time PCR System (Bio-Rad, Milan, Italy). Each run included a no-RNA negative control and melting-curve analysis of primer pairs. RNA expression was analyzed with CFX Manager software (Bio-Rad CFX Maestro 1.1 (Version: 4.1.2433.1219)). Data were normalized to *Rps18* for testis/TM3 cells and *Cyclophilin* for sperm cells. Normalized fold expression (nfe) of circRNAs was calculated using the 2^^−ΔΔCt^ method.

Results were reported as mean nfe ± SEM. The online tool Primer-BLAST (http://www.ncbi.nlm.nih.gov/tools/primer-blast/, accessed on 15 January 2025) was used to design murine primers. Primer sequences are reported in Table 1.

### 2.13. Functional Annotation for circRNA/miRNA and Target miRNA Interaction

The circRNA/miRNA interaction for circNFAT5 was predicted with Arraystar’s miRNA target prediction software and circATLAS 3.0 (https://ngdc.cncb.ac.cn/circatlas accessed on 10 March 2025). The miRNA targets were obtained by Diana TarBase 8.0 (http://www.microrna.gr/tarbase). CircRNA/miRNA/Target networks (ceRNETs) building was performed by using Bisogenet plug-in of Cytoscape (www.cytoscape.org).

### 2.14. Cell Proliferation Assays

The CyQUANT™ XTT Cell Viability Assay (Invitrogen, Thermo Fisher Scientific, Waltham, MA, USA) was used to evaluate cell proliferation according to the manufacturer’s instructions. Briefly, TM3 cells in vitro treated with a siRNA against NFAT5 (n = 3 for each experimental condition) cells were plated in 96-well plates, and transfection (or cell treatment) was performed as described above. The cell growth was evaluated by adding 70 µL of XTT/electron coupling reagent to each well. After 1 h of incubation at 37 °C, the absorbance was measured at 450 nm with a reference wavelength of 650 nm using the GloMAX discover system (Promega, Madison, WI, USA).

### 2.15. Nuclear and Cytoplasmic Protein Extraction

Protein samples were extracted from nuclear and cytoplasmic fractions. Briefly, the untreated TM3 cells and TM3 cells in vitro treated with H_2_O_2_ Aliquots (n = 3 for each experimental condition) were resuspended in 1 mL of PBS and centrifuged at 500× *g* for 5 min (4 °C). The cell pellet was resuspended in 50 µL of solution A (10 mM of Hepes pH 7.9, 1.5 mM of MgCl_2_, 10 mM of KCl, 1 mM of Na_3_VO_4_, 1 mM of PMSF, 1 mg/mL Leupeptin), incubated on ice for 15 min, mixed by vortexing, and then centrifuged at 10,000× *g* for 15 min (4 °C). The supernatant containing the cytoplasmic protein fraction was collected. The pellet was washed twice with solution A and then resuspended in 50 µL of solution B (20 mM of Hepes pH 7.9, 25% glycerol, 420 nm of NaCl, 1.5 mM of MgCl_2_, 0.2 mM of EDTA pH 8.0, 1 mM of Na_3_VO_4_, 1 mM of PMSF, 1 mg/mL Leupeptin), incubated for 20 min on ice, mixed by vortexing, and then centrifuged at 10,000× *g* for 15 min (4 °C). The supernatant containing the nuclear protein fraction was collected. The amount of nuclear and cytoplasmic proteins was assessed using the Lowry assay.

### 2.16. Caspase 3/7 Activity Assay

Caspase-3/7 activity was measured using the Caspase-Glo^®^ 3/7 Assay (Promega, Madison, WI, USA) following the manufacturer’s instructions. Cells were seeded in quadruplicate in 96-well plates, allowed to adhere, and treated with increasing concentrations of H_2_O_2_ (0, 100, 200, 400 µM) for 4, 8, or 24 h. At the end of treatment, luminescence was measured using a GloMAX^®^ Discover microplate reader (Promega, Madison, WI, USA). Caspase-3/7 activity data were normalized to cell viability using the CyQUANT™ XTT Cell Viability Assay (Invitrogen, Thermo Fisher Scientific, Waltham, MA, USA). Data were expressed relative to untreated controls. Based on these analyses, the condition of 400 μM H_2_O_2_ for 24 h was selected for subsequent experiments.

### 2.17. Cell Immunofluorescence Analysis

Untreated TM3 cells and TM3 cells *in vitro* treated with H_2_O_2_ aliquots (n = 3 for each experimental condition) were fixed in 4% paraformaldehyde (sc-281692; Santa Cruz Biotechnology, Heidelberg, Germany) for 20 min at RT and permeabilized with 0.1% Triton X-100 (X100; Sigma-Aldrich, Milano, Italy). After blocking step with a blocking solution containing 10% donkey serum (ab7475; Abcam, Cambridge, UK), the cells were incubated ON at 4 °C with anti-NFAT5 primary antibody (sc-398171, Santa Cruz Biotechnology, Heidelberg, Germany) diluted 1:100. Following three washes in PBS, a Cy5 (111-175-144; Jackson ImmunoResearch, Cambridge, UK) conjugate secondary antibody diluted 1:200 was used for 1 h at 37 °C. Nuclei were labeled with DAPI (D9542; Sigma-Aldrich, Milano, Italy). Slides were analyzed under an optical microscope (Leica DM 5000 B + CTR 5000; Leica Microsystems, Wetzlar, Germany) with a UV lamp. Images were viewed with IM 1000 software (version 4.7.0; Leica Microsystems, Wetzlar, Germany) and captured by using Leica DFC320 R2 digital camera.

### 2.18. Statistical Analysis

The sample size (n = 6 per group) was validated by a power analysis using G*Power software (v3.1.9.7). Based on a predicted large effect size, a total n = 18 was determined to provide a power of 0.80 with a significance level of 0.05. This cohort size is also in accordance with the 3Rs principles for animal welfare.

Data are presented as mean ± SEM. The normality of data distribution and the homogeneity of variance were assessed using the Shapiro–Wilk and Levene’s tests, respectively. Statistical significance was determined by one-way ANOVA followed by Tukey’s post hoc test for multiple comparisons when evaluating differences among CTRL, HFD, and F1H groups. For comparisons between two experimental groups, an unpaired Student’s *t*-test was applied. For in vitro assays using the TM3 cell line, experiments were performed in triplicate and analyzed *via* unpaired Student’s *t*-test. Differences were considered statistically significant at *p* < 0.05 or *p* < 0.01.

## 3. Results

### 3.1. High-Fat Diet Affects Testis Oxidative Status, Leydig Cell Survival, and Steroidogenic Activity

**Figure 1:**
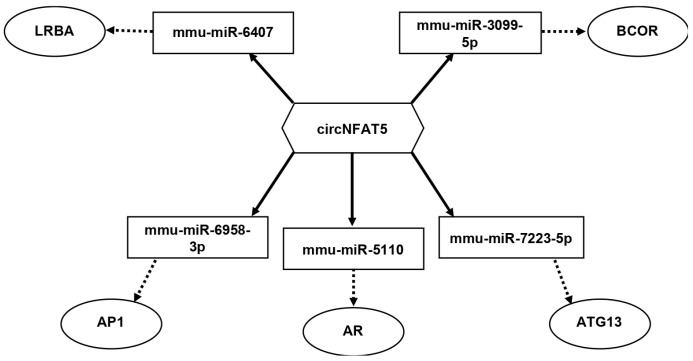
CircNFAT5 regulatory ceRNET modulating LC functional activities; *circNFAT5* tethers a group of miRNAs and mRNAs as targets, all involved in the regulation of LCs and apoptotic pathways. Networks were built using Cytoscape. Hexagonal and rectangular symbols represent circRNAs and miRNAs, respectively. The arrow indicates the tethering activity of circRNAs toward miRNAs, while the dotted arrow indicates the pathways downstream of the miRNAs.

Considering the established role of NFAT2 in regulating LC apoptosis [21] and the dysregulation of circNFAT5 previously observed in HFD SPZ [19], we hypothesized that sperm-associated circNFAT5 might mirror alterations in LC-related molecular pathways within the testis. Based on this, we bioinformatically constructed a competing endogenous RNA network (ceRNET) for *circNFAT5* to identify its potential regulatory role in modulating LC functional activities and characterized its expression in HFD testis at both transcriptional and protein level. The bioinformatic analysis provided the top five *circNFAT5*-miRNA targets: mmu-miR-6407; mmu-miR-3099-5p; mmu-miR-7223-5p; mmu-miR-5110; mmu-miR-6958-3p (Figure 1). Within the set of miRNA targets, several transcripts involved in the regulation of LCs and apoptotic pathways were identified, including: (i) BCOR, that influences apoptotic pathways by enhancing BCL-6-mediated transcriptional programs [26]; (ii) LRBA, a factor modulating apoptotic and survival pathways [27]; (iii) AP1, a critical modulator of LC proliferation and steroidogenic [28]; (iv) AR, the androgen receptor fundamental for male reproductive integrity [2]; and (v) ATG13, that acts as regulator of LC autophagy [29].

**Figure 2:**
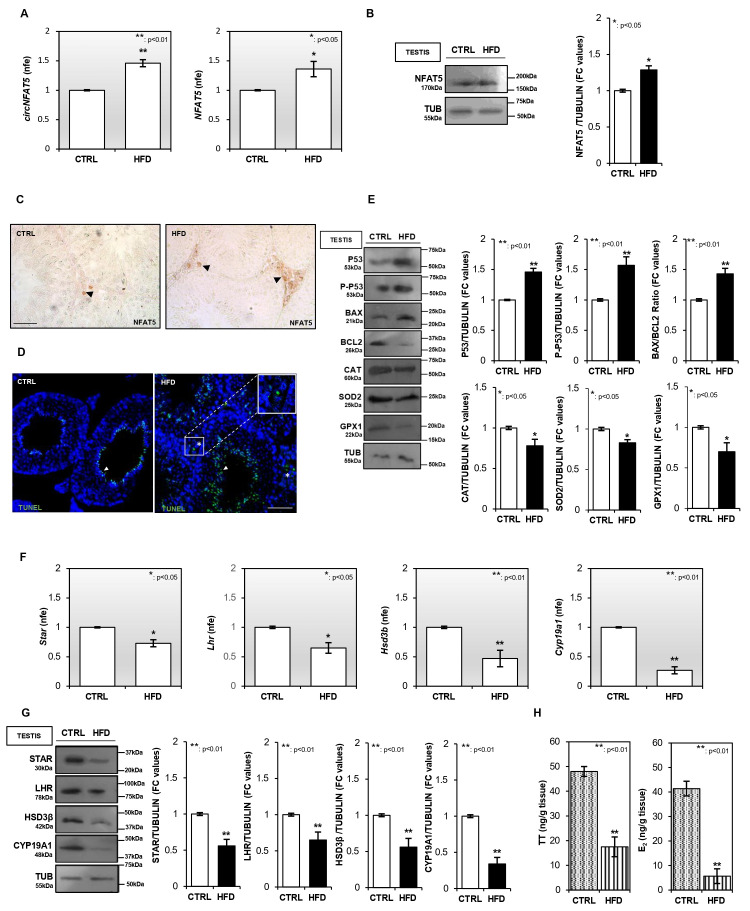
HFD exposure promotes Leydig cell apoptosis. (**A**) Expression analysis of *circNFAT5* and *NFAT5* in CTRL and HFD testes. (**B**) Western blot analysis of NFAT5 protein in CTRL and HFD testes. (**C**) Immunocytochemistry analysis of NFAT5 in Bouin’s fixed testis sections (7 µm) of CTRL and HFD testes. The NFAT5 protein localization in LC is indicated by black arrows. Scale bar, 50 µm. (**D**) TUNEL assay in CTRL and HFD testis sections (7 µm). The white arrows shows spermatid TUNEL-positive (green) cells while the white asterisk shows TUNEL-positive LCs. Scale bar, 50 µm. (**E**) Western blot analysis of P53, P-P53, BAX, BCL2, CAT, SOD2, and GPX1 proteins in CTRL and HFD testes. (**F**) Expression analysis of *Star*, *Lhr*, *Hsd3b*, and *Cyp19a1* in CTRL and HFD testes. (**G**) Western blot analysis of STAR, LHR, HSD-3β, and CYP19A1 proteins in CTRL and HFD testes. (**H**) Analysis of testosterone (TT) and 17-β-estradiol (E_2_) intratesticular content (as ng/g) in CTRL and HFD mice. Data are expressed as ng/g tissue and reported as mean value ± S.E.M; **: *p* < 0.01. Western blot signals were quantified by densitometry analysis, normalized against TUBULIN, expressed in fold change (FC) OD values, and reported as mean ± S.E.M; ** *p* < 0.01; * *p* < 0.05. One-step qPCR data were normalized using *Rps18*, expressed as fold expression (nfe), and reported as mean value ± S.E.M; ** *p* < 0.01; * *p* < 0.05.

Relative to the CTRL group, one-step qPCR analysis revealed a significant (*p* < 0.01; *p* < 0.05) increase in both *circNFAT5* and in its linear counterpart (*NFAT5*) in HFD testis (Figure 2A). Similarly, NFAT5 protein content was significantly higher (*p* < 0.05) in HFD compared with CTRL testis (Figure 2B). The morphological characterization of NFAT5 protein was performed in CTRL and HFD testis by immunohistochemistry (IHC) staining, revealing a strong signal, especially in the interstitial compartment (Figure 2C).

In order to reveal the triggering of apoptotic pathways in HFD LCs, we performed morphological and molecular analyses on testes. Specifically, a terminal deoxynucleotidyl transferase dUTP nick end labeling (TUNEL) assay was carried out in HFD testis cross-sections in comparison with CTRL ones. As shown in Figure 2D, a physiological TUNEL-positive signal was detected in CTRL testes, mainly at the spermatid level. Relative to the CTRL counterpart, HFD testis sections exhibited a higher frequency of TUNEL-positive spermatid cells. Moreover, in HFD testis, a clear positive labeling was also detected in the interstitial compartment, specifically localized in LCs, suggesting that HFD exposure promoted LC apoptosis (Figure 2D). Consistently, Western blot analysis revealed a significant (*p* < 0.01) increase in P53 content and in its relative phosphorylated form (P-P53) in HFD compared with CTRL testis. The levels of proapoptotic (BAX) and antiapoptotic (BCL2) modulators appeared higher and lower in HFD testis, respectively. Consequently, a significant increase (*p* < 0.01) in BAX/BCL2 protein ratio was observed in HFD testis (Figure 2E), confirming a shift toward a proapoptotic state under HFD conditions. To further investigate whether the proapoptotic shift observed in HFD testis was associated with alterations in the antioxidant defense system, the content of key antioxidant enzymes was assessed. Specifically, the levels of catalase (CAT), superoxide dismutase 2 (SOD2), and glutathione peroxidase 1 (GPX1) were analyzed in CTRL and HFD testes by Western blot analysis. All antioxidant markers were significantly (*p* < 0.05) reduced in HFD compared with CTRL testes (Figure 2E), confirming an increased oxidative stress in HFD conditions potentially sustaining the characterized proapoptotic state.

Given the observed oxidative stress and apoptotic phenotype, we investigated LC functionality by analyzing the expression levels of STAR, LHR, HSD-3β, and CYP19A1 makers by one-step qPCR and Western blot analyses. A significant (*p* < 0.01) reduction in *Star*, *Lhr*, *Hsd3b*, and *Cyp19a1* transcripts was observed in HFD compared with CTRL testis (Figure 2F). Accordingly, the protein content of all LC markers investigated was significantly (*p* < 0.01) lower in HFD than CTRL testis (Figure 2G), suggesting an impaired LC steroidogenic activity in HFD mice. In agreement with this assumption, intra-testicular dosage of testosterone (TT) and 17-β-estradiol (E_2_) performed by LC-MS analysis revealed a significant reduction in both steroidogenic hormones in HFD compared with CTRL testis (Figure 2H). Altogether, these findings delineate a profile of HFD-induced testicular dysfunction defined by oxidative imbalance, increased cell death, and impaired steroidogenesis. These results prompted us to investigate whether NFAT5 acts as a key molecular mediator in regulating LC apoptosis.

### 3.2. NFAT5 Promotes Leydig Cell Apoptosis via Its Nuclear Shuttling

**Figure 3:**
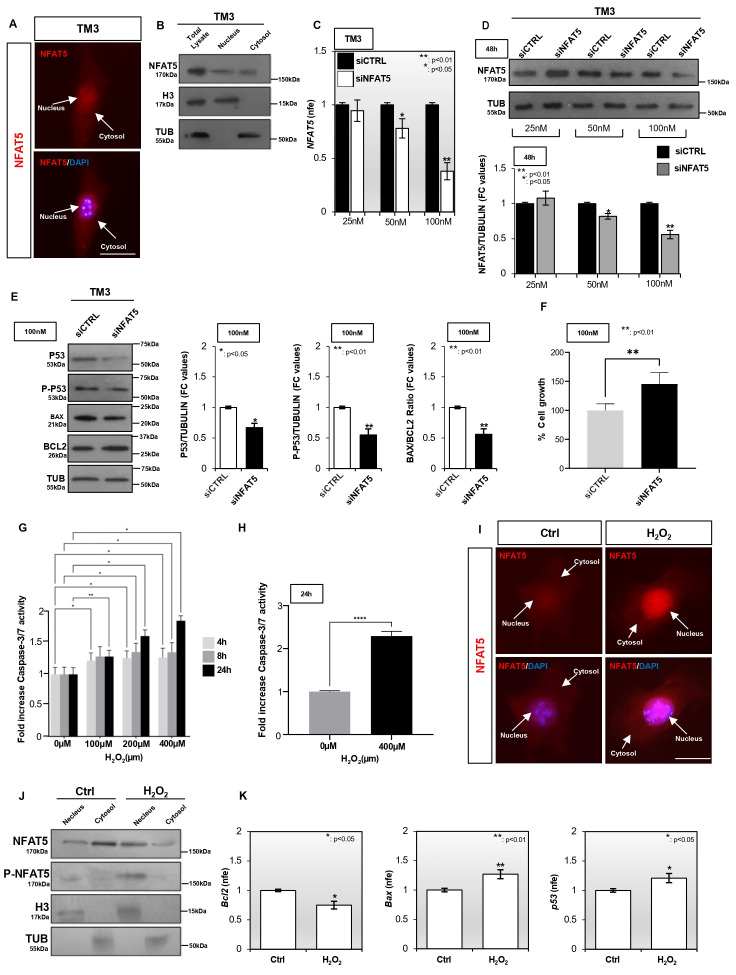
NFAT5 nuclear localization promotes Leydig cell apoptosis. (**A**) NFAT5 immunofluorescence analysis (red) in TM3 cells. Nuclei are labeled with DAPI (blue); scale bar, 15 μm. (**B**) Western blot analysis of NFAT5, histone H3, and TUBULIN proteins in TM3 nuclear and cytosolic compartments. (**C**) Expression analysis of *NFAT5* transcript in TM3 cells transfected with siRNA Universal Negative Control (siCTRL) or siNFAT5 at 25 nM, 50 nM, and 100 nM for 48 h. (**D**) Western blot analysis of NFAT5 protein in TM3 transfected with siRNA Universal Negative Control (siCTRL) or siNFAT5 at 25 nM, 50 nM, and 100 nM for 48 h. (**E**) Western blot analysis of P53, P-P53, BAX, and BCL2 proteins in TM3 cells transfected with siRNA Universal Negative Control (siCTRL) or siNFAT5 at 100 nM for 48 h. (**F**) Cell growth analysis in TM3 cells transfected with siRNA Universal Negative Control (siCTRL) or siNFAT5 at 100 nM for 48 h. Data were normalized against the CTRL group, expressed as percentage of cell growth, and reported as mean ± S.E.M; ** *p* < 0.01. (**G**,**H**) Quantification of Caspase-3/7 activity in TM3 cells following in vitro treatment with H_2_O_2_ (0 µM; 100 µM; 200 µM; 400 µM) at different time points (4 h; 8 h; 24 h). Data were reported as mean ± S.E.M; * *p* < 0.05, ** *p* < 0.01, **** *p* < 0.0001. (**I**) NFAT5 immunofluorescence analysis (red) in TM3 cells CTRL and in vitro treated with H_2_O_2_ (400 µM/24 h). Nuclei are labeled with DAPI (blue); scale bar, 15 μm. (**J**) Western blot analysis of NFAT5, P-NFAT5, histone H3, and TUBULIN proteins in nuclear and cytosolic compartments of TM3 cells CTRL and in vitro treated with H_2_O_2_ (400 µM/24 h). (**K**) Expression analysis of *Bcl2*, *Bax*, and *p53* transcripts in TM3 cells CTRL and in vitro treated with H_2_O_2_ (400 µM/24 h). One-step qPCR data were normalized using *Rps18*, expressed as fold expression (nfe), and reported as mean value ± S.E.M; ** *p* < 0.01; * *p* < 0.05.

To decipher the NFAT5-driven molecular pathways underlying LC apoptosis, we established two complementary in vitro strategies using the TM3 immortalized LC line. First, we employed RNA interference-mediated *NFAT5* silencing to assess its impact on cell viability. Subsequently, we performed an in vitro apoptosis induction assay to further elucidate the role of NFAT5 transcriptional activity in this regulatory framework. Considering that NFAT5 transcriptional activity depends on its phosphorylation-mediated subcellular localization [30], we morphologically and molecularly characterized NFAT5 distribution in TM3 cell compartments. Immunofluorescence analysis showed a widely NFAT5 signal distributed in both the cytoplasmic and nuclear compartments (Figure 3A). Protein lysates collected from nuclear and cytosolic compartments were analyzed to confirm NFAT5 subcellular distribution. The quality of cell fractioning was evaluated by assessing histone H3 and TUBULIN as representative nuclear and cytosolic markers, respectively (Figure 3B). In comparison to the total protein content, Western blot analysis showed a dual subcellular localization of NFAT5, both the nucleus and cytosol, in TM3 cells, at a lesser extent in the nucleus (Figure 3B).

To verify the involvement of NFAT5 in modulating cell apoptotic pathway, a specific pool of siRNA for *NFAT5* mRNA (siNFAT5) was transfected in TM3 cells at different doses (25 nM; 50 nM; 100 nM) for 48 h. The one-step qPCR analysis showed a significant downregulation of *NFAT5* transcript at the doses of 50 nM (*p* < 0.05) and 100 nM (*p* < 0.01) in comparison with the relative negative control (siCTRL), although a more efficient silencing occurred at the 100 nM dose (Figure 3C). Accordingly, Western blot analysis confirmed a more significant (*p* < 0.01) reduction in NFAT5 protein at the 100 nM dose (Figure 3D). Based on these results, we performed the downstream investigations choosing the more silencing 100 nM dose. Relative to the siCTRL group, Western blot analysis revealed a significant reduction in P53 (*p* < 0.05) and P-P53 (*p* < 0.01) protein contents, as well as in BAX/BCL2 protein ratio, in TM3 cells transfected with siNFAT5 (Figure 3E). In agreement with molecular data, a significant upregulation of TM3 cell growth percentage (*p* < 0.01) was observed in the siNFAT5 compared to the siCTRL experimental group (Figure 3F), demonstrating that the *NFAT5* silencing promoted the establishing of an antiapoptotic phenotype.

To confirm this assumption, we reversed the experimental strategy by establishing an in vitro apoptosis-induction assay. Specifically, TM3 cells were in vitro treated with increasing doses of H_2_O_2_ (0 µM; 100 µM; 200 µM; 400 µM) at different time points (4 h; 8 h; 24 h) and subsequently processed to quantify Caspase3/7 activation in order to determine the experimental condition that most efficiently triggered apoptotic activation. Although a significant Caspase3/7 activation was detected across multiple concentrations, the highest apoptotic efficiency was achieved at the 400 µM dose of H_2_O_2_ administered for 24 h (Figure 3G). Hence, we performed the downstream investigations choosing the most proapoptotic condition (400 µM at 24 h). After confirming Caspase3/7 activation (Figure 3H), NFAT5 immunofluorescence analysis was carried out in CTRL and H_2_O_2_ in vitro treated TM3 cells. As shown in Figure 3I, a pronounced increase in NFAT5 nuclear signal was detected following H_2_O_2_ in vitro treatment, indicating that the activation of the apoptotic pathway correlated with NFAT5 shuttling in the nuclear compartment. Western blot analysis of protein lysates collected from nuclear and cytosolic compartments of CTRL and H_2_O_2_ in vitro treated TM3 cells showed an increased NFAT5 nuclear content in the H_2_O_2_ experimental group, confirming an enhanced NFAT5 shuttling upon the apoptosis induction (Figure 3J). Accordingly with this assumption, Western blot analysis of the NFAT5 phosphorylated form (P-NFAT5) revealed a markedly enhanced nuclear signal in H_2_O_2_ in vitro treated cells when compared with CTRL ones, although a faint cytosolic signal was detectable in both experimental conditions (Figure 3J). Lastly, to determine whether NFAT5 nuclear accumulation could modulate the gene expression of apoptotic regulators, the transcript levels of *Bcl2*, *Bax*, and *P53* were investigated by one-step qPCR analysis. Compared with the CTRL group, a significant (*p* < 0.05) reduction in *Bcl2*, together with increased *Bax* and *p53* expression levels, was observed following H_2_O_2_ in vitro treatment (Figure 3K), confirming that NFAT5 nuclear shuttling correlated with the activation of proapoptotic genes. Overall, these results demonstrate that NFAT5 activation orchestrates a proapoptotic program within LCs. Based on these mechanistic insights, we next sought to determine whether the NFAT5 dysregulation observed in HFD mice could be transmitted to the offspring, potentially compromising intergenerational reproductive health.

### 3.3. Intergenerational Recovery of NFAT5 Signature and Mitigation of Oxidative Stress and Testicular Apoptosis in F1H Progeny

Beyond its detrimental effects on testicular steroidogenesis—which compromises sperm quality and drives male infertility—paternal obesity has been increasingly linked to adverse developmental outcomes in progeny, including increased susceptibility to obesity, metabolic dysfunction, and cognitive impairments [31,32].

Hence, we generated the male progeny (F1H) derived from HFD fathers and performed testicular and morphological investigations to evaluate a putative paternal intergenerational transmission of testicular damage. The F1H offspring characterization was conducted relative to both HFD and CTRL fathers in order to determine whether any physiological recovery could occur.

**Figure 4:**
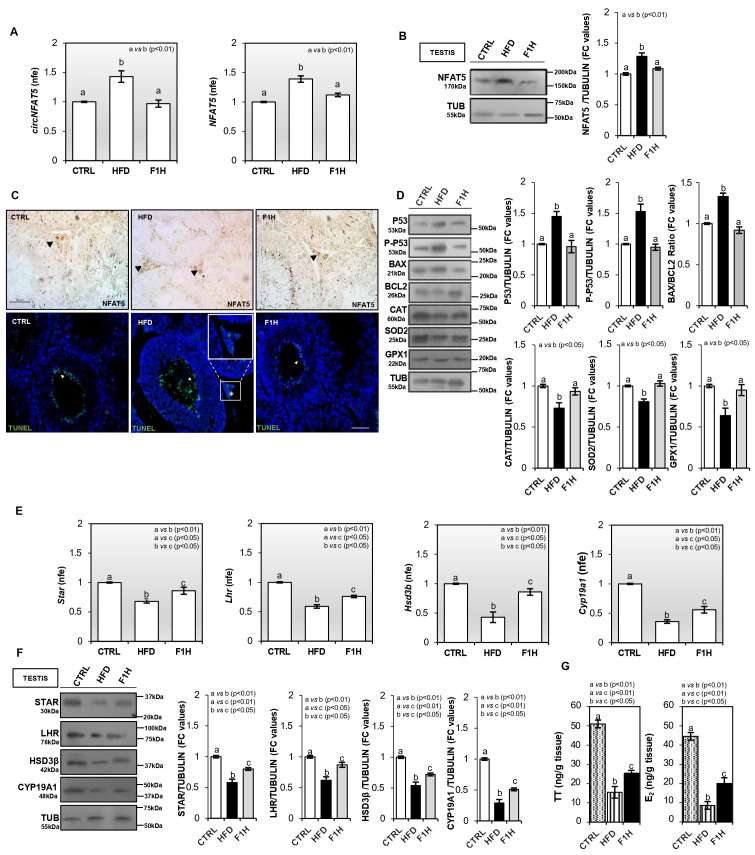
Leydig cell steroidogenic defects are transmitted to HFD offspring. (**A**) Expression analysis of *circNFAT5* and *NFAT5* in CTRL, HFD, and F1H testes. (**B**) Western blot analysis of NFAT5 protein in CTRL, HFD, and F1H testes. (**C**) Immunocytochemistry analysis of NFAT5 in Bouin’s fixed testis sections (7 µm) and TUNEL assay in CTRL, HFD, and F1H testis sections (7 µm). The NFAT5 protein localization in LC is indicated by black arrows. The white arrows show spermatid TUNEL-positive (green) cells while the white asterisk shows TUNEL-positive LCs. Scale bar, 50 µm. (**D**) Western blot analysis of P53, P-P53, BAX, BCL2, CAT, SOD2, and GPX1 proteins in CTRL, HFD, and F1H testes. (**E**) Expression analysis of *Star*, *Lhr*, *Hsd3b*, and *Cyp19a1* CTRL, HFD, and F1H testes. (**F**) Western blot analysis of STAR, LHR, HSD-3β, and CYP19A1 proteins in CTRL, HFD, and F1H testes. (**G**) Analysis of testosterone (TT) and 17-β-estradiol (E_2_) intratesticular content (as ng/g) in CTRL, HFD, and F1H testes mice. Data were expressed as ng/g tissue and reported as mean value ± S.E.M. Western blot signals were quantified by densitometry analysis, normalized against TUBULIN, expressed in fold change (FC) OD values, and reported as mean ± S.E.M. One-step qPCR data were normalized using *Rps18*, expressed as fold expression (nfe), and reported as mean value ± S.E.M. One-step qPCR data were normalized using *Rps18*, expressed as fold expression (nfe), and reported as mean value ± S.E.M. Experimental groups with statistically significant differences were indicated by different letters.

Relative to the CTRL group, the one-step qPCR analysis confirmed a significant (*p* < 0.01) increase in both *circNFAT5* and linear *NFAT5* in HFD testis. However, in F1H testis the expression levels of *circNFAT5* and linear *NFAT5* were comparable to CTRL values (Figure 4A). Similarly, Western blot analysis confirmed the NFAT5 protein increase (*p* < 0.01) in HFD when compared to CTRL testis, whereas no significant changes occurred among CTRL and F1H experimental groups (Figure 4B). In agreement with molecular data, the morphological characterization of NFAT5 protein in CTRL, HFD, and F1H testis cross-sections performed by IHC staining confirmed a less pronounced NFAT5 immunosignal, accomplished with a reduced frequency of labeled LCs, in F1H testis (Figure 4C), suggesting that the recovery of the NFAT5 signature in HFD progeny was potentially associated with the recovery of LC apoptosis.

In order to confirm this hypothesis, we performed a TUNEL assay in testis cross-sections. As shown in Figure 4C, a physiological TUNEL-positive signal was detected in CTRL testes, mainly at the spermatid level. The immunofluorescence analysis confirmed that HFD testis sections exhibited a higher frequency of TUNEL-positive spermatid cells, accomplished with clear positive labeling of the interstitial compartment specifically localized to LCs. Conversely, the F1H cross-sections showed an immunofluorescence pattern more comparable with the CTRL one, characterized by few labeled spermatids and the absence of an LC signal (Figure 4C). Accordingly, Western blot analysis revealed a significant (*p* < 0.01) reduction to CTRL values in P53 and P-P53 protein contents in F1H compared with HFD testis (Figure 4D). A similar trend was also observed for BAX/BCL2 protein ratio (Figure 4D), confirming a recovery of the apoptotic phenotype in F1H LCs. In agreement with apoptosis recovery, a significant (*p* < 0.05) increase to CTRL values in CAT, SOD2, and GPX1 expression occurred in F1H testis (Figure 4D), confirming a closer functional relationship between testicular oxidative stress and apoptotic processes. Then, we verified LC functionality by analyzing the gene expression of STAR, LHR, HSD-3β, and CYP19A1 makers by one-step qPCR and Western blot analyses. A significant (*p* < 0.01) reduction in *Star*, *Lhr*, *Hsd3b*, and *Cyp19a1* transcripts was confirmed in HFD when compared with CTRL testis (Figure 4E). Despite a significant (*p* < 0.05) increase compared with HFD values, the F1H testis did not show a complete recovery of these transcripts to the physiological CTRL levels (Figure 4E). Accordingly, the protein content of all LC markers investigated was significantly (*p* < 0.01) lower in HFD than in CTRL testes, and F1H progeny showed only a partial recovery, without reaching physiological CTRL values (Figure 4F). Similarly, although intra-testicular LC-MS quantification of TT and E2 revealed a significant (*p* < 0.05) increase in F1H compared with HFD values, the hormone concentrations did not fully return to the physiological range detected in CTRL testes (Figure 4G). In summary, while the F1H progeny displays a remarkable recovery of the testicular NFAT5-mediated apoptotic signature, the steroidogenic machinery remains significantly compromised. This divergence suggests that, despite a partial physiological restoration within the testis, a “molecular memory” of paternal HFD-induced damage persists. Given that steroidogenic impairment often translates into post-testicular defects, we next investigated whether this lingering dysfunction correlates with alterations in the morpho-functional parameters and epigenetic cargo of epididymal SPZ.

### 3.4. Dysregulated Epididymal NFAT5 Content Is Associated with Altered Sperm Parameters and circRNA Cargo in HFD Progeny

Given the partially restored yet still compromised steroidogenic profile observed in the progeny of HFD mice, we next evaluated the morpho-functional parameters of SPZ collected from F1H mice in comparison with their HFD fathers and CTRL. Analyses were performed on SPZ derived from the *cauda* epididymis, as this anatomical compartment contains the functionally mature sperm population responsible for fertilization.

**Figure 5:**
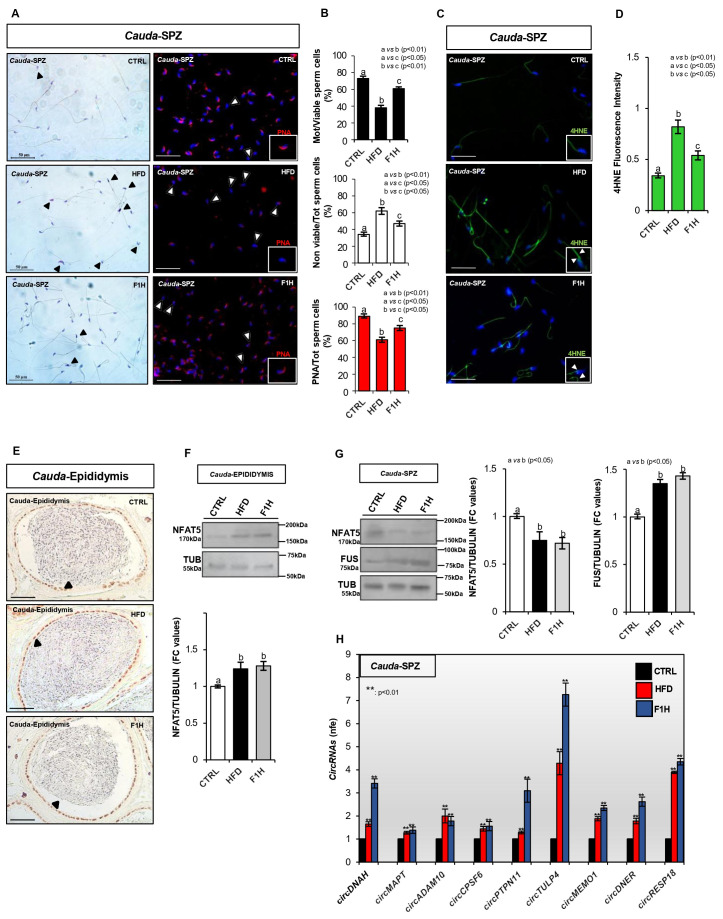
NFAT5 impairment is associated with intergenerational inheritance of abnormal sperm circRNA cargo. (**A**) H&E and PNA (red) immunofluorescence analyses on *cauda* SPZ collected from CTRL, HFD, and F1H mice. Anomalous sperm morphology in H&E staining is indicated by black arrowheads. PNA null sperm heads are indicated by white arrowheads. Scale bar, 50 μm. (**B**) Evaluation of sperm motility, viability, and PNA positivity staining in *cauda* SPZ collected from CTRL, HFD, and F1H mice. Sperm motility and viability are expressed as the percentage of motile/live SPZ and non-viable/total SPZ, respectively. PNA positivity is expressed as percentage of positive cell. Experimental groups with statistically significant differences are indicated by different letters. (**C**) Immunofluorescent analysis of 4HNE on *cauda* SPZ collected from CTRL, HFD, and F1H mice. White arrows indicate 4-HNE localization in the posterior part of the sperm head and in the flagellum. (**D**) Histogram showing the quantification of 4HNE intensity using ImageJ software. Values are expressed as mean fluorescence intensity ± SEM. Experimental groups with statistically significant differences are indicated by different letters. (**E**) Immunocytochemistry analysis of NFAT5 in Bouin’s fixed *cauda* epididymis sections (7 µm) of CTRL, HFD, and F1H mice. NFAT5 protein localization is indicated by black arrows. Scale bar, 50 µm. (**F**) Western blot analysis of NFAT5 protein in *cauda* epididymis of CTRL, HFD, and F1H. (**G**) Western blot analysis of NFAT5 and FUS proteins in *cauda* SPZ of CTRL, HFD, and F1H mice. (**H**) Expression analysis of *circDNAH*, *circMAPT*, *circADAM10*, *circCPSF6*, *circTULP4*, *circMEMO1*, *circPTPN11*, *circDNER*, *circRESP18* in CTRL, HFD, and F1H *cauda* SPZ. Western blot signals were quantified by densitometry analysis, normalized against TUBULIN, expressed in fold change (FC) OD values, and reported as mean ± S.E.M; experimental groups with statistically significant differences are indicated by different letters. One-step qPCR data were normalized using *Cyclophilin*, expressed as fold expression (nfe), and reported as mean value ± S.E.M; ** *p* < 0.01.

Sperm morphological characterization carried out by H&E staining highlighted several anomalies in *cauda* SPZ collected from HFD and F1H mice in terms of head shaping defects, while no defects were observed in sperm tails (Figure 5A). In addition, the evaluation of sperm functional parameters highlighted a significant (*p* < 0.01) reduction in the percentage of motile SPZ in HFD *cauda* SPZ (Figure 5B). Although a significant (*p* < 0.05) increase in the percentage of motile SPZ was observed in F1H when compared with HFD *cauda* SPZ, this increase did not reach physiological values (Figure 5B). Accordingly, a similar trend was observed in the percentage of non-viable SPZ. A significant (*p* < 0.01) increase in non-viable SPZ occurred in HFD when compared with the CTRL experimental group. Although the percentage of non-viable SPZ was significantly (*p* < 0.05) lower in F1H than HFD *cauda* SPZ, the values were not recovered to physiological levels (Figure 5B).

The acrosome morphology evaluated by peanut lectin PNA immunofluorescence analysis revealed a PNA signal well confined to the half anterior part of the sperm head consisting of the acrosomal region in CTRL *cauda* SPZ. Conversely, a high frequency of SPZ showing a PNA null signal was observed in both HFD and F1H mice (Figure 5A). In agreement, relative to CTRL group, the sperm count using acrosomal PNA localization as the inclusive analysis parameter showed a significant reduction (*p* < 0.01) in the percentage of SPZ with positive PNA signal in the HFD experimental group (Figure 5B), suggesting a potential early acrosome reaction (AR) onset. Although a significant (*p* < 0.05) increase in the percentage of PNA-positive SPZ was observed in F1H when compared with HFD *cauda* SPZ, this increase did not reach physiological values (Figure 5B).

Given the incomplete restoration of sperm motility observed in F1H mice, we performed an immunofluorescence analysis of 4-HNE, a key marker of lipid peroxidation known to impair flagellar movement and inactivate antioxidant enzymatic systems [33]. Regardless of the experimental condition, the 4HNE signal was mainly detected in sperm tails, despite the fact that a weak signal was also observed in sperm heads (Figure 5C). The quantitative fluorescence intensity analysis confirmed a significant (*p* < 0.01) increase in 4HNE signal in HFD when compared with CTRL *cauda* SPZ (Figure 5D). Conversely, in F1H *cauda* SPZ, the 4HNE fluorescence intensity was significantly (*p* < 0.05) reduced compared with HFD fathers but did not reach the physiological CTRL values (Figure 5D), confirming that HFD progeny only partially recovered the oxidative status and sperm physiological morpho-functional phenotype.

Before ejaculation, SPZ reside in a physiologically hyperosmotic epididymal environment [34,35,36]. However, the sperm cells are highly sensitive to osmotic fluctuations, and both hypo- and hyperosmotic conditions can rapidly compromise their motility and overall functional integrity [37]. Interestingly, in multiple cell systems, NFAT5 plays a pivotal role in osmotic stress adaptive response, particularly under hypertonic conditions, by controlling the expression of osmo-protective genes that increase intracellular osmolytes and preserve cell function [30]. Based on this background, we morphologically and molecularly characterized NFAT5 protein in *cauda* epididymis derived from CTRL, HFD, and F1H mice in order to investigate a potential NFAT5-dependent regulatory role in epididymal sperm maturation.

The IHC analysis performed in *cauda* epididymal cross-sections showed a well-defined nuclear NFAT5 localization in the epididymal cells of all experimental groups investigated (Figure 5E), although a more intense signal was detected in both HFD and F1H experimental groups. Accordingly, Western blot analysis showed a significant (*p* < 0.05) NFAT5 increase in HFD and F1H when compared with CTRL *cauda* epididymis (Figure 5F). Considering that epididymal-derived exosomes (epididymosomes) contribute to the enrichment of sperm protein cargo [36], we investigated NFAT5 content in *cauda* SPZ collected from CTRL, HFD, and F1H mice by Western blot analysis. As shown in Figure 5G, a significant (*p* < 0.05) reduction in NFAT5 protein was observed in HFD and F1H when compared with CTRL *cauda* SPZ, suggesting that despite the NFAT5 epididymal enrichment observed in HFD and F1H mice, a conceivable impaired epididymal delivery to SPZ occurred.

In several cellular systems, osmotic stress may drive epigenetic and transcriptional reprogramming, providing a mechanism by which cells can adapt their function under dynamic osmotic conditions [38,39]. Based on this premise, in order to determine whether epididymal NFAT5 impairment could be associated with a deregulation of the sperm circRNA epigenetic profile *via* paternal intergenerational transmission, we explored the epigenetic responsiveness of the sperm circRNA cargo. Specifically, we analyzed spermatic fused in sarcoma (FUS) content and a set of spermatic circRNAs previously reported to be upregulated in HFD mice and generated through an enhanced endogenous backsplicing driven by FUS protein [19]. Relative to the CTRL group, the Western blot analysis showed a significant increase (*p* < 0.01) in FUS protein in both HFD and F1H *cauda* SPZ (Figure 5G), suggesting enhanced backsplicing activity. The expression analysis of 9 circRNAs (*circDNAH*, *circMAPT*, *circADAM10*, *circCPSF6*, *circTULP4*, *circMEMO1*, *circPTPN11*, *circDNER*, *circRESP18*), previously reported to be upregulated in HFD sperm [19], was performed in CTRL, HFD, and F1H *cauda* SPZ by one-step qPCR analysis. Relative to CTRL, a significant increase (*p* < 0.01) in all circRNAs investigated was confirmed in HFD *cauda* SPZ (Figure 5H). Interestingly, an enhanced upregulated trend also occurred in F1H cauda SPZ (Figure 5H), demonstrating that the impaired sperm circRNA profile was amplified in HFD progeny. In conclusion, these findings demonstrate that paternal HFD disrupts epididymal-to-sperm NFAT5 delivery, leading to an amplified dysregulation of the circRNA cargo in offspring. This suggests that the epididymal NFAT5-FUS axis serves as a key driver for the intergenerational transmission of sperm epigenetic defects and compromised reproductive fitness.

## 4. Discussion

The present study elucidates a previously uncharacterized NFAT5-dependent molecular axis underlying LC dysfunction and spermatic epigenetic aberrations. By integrating *in vivo*, in vitro, and intergenerational approaches, our findings demonstrate that obesity-driven disruption of NFAT5 homeostasis concomitantly impairs testicular oxidative balance, LC survival, and steroidogenic competence. Furthermore, this dysregulation extends to epididymal maturation, ultimately driving an aberrant remodeling of the sperm circRNA cargo in both HFD-fed fathers and their progeny. Our results reveal that NFAT5 is selectively expressed in LCs. Under HFD conditions, NFAT5 overexpression was associated with increased LC apoptosis and impaired steroidogenic competence, leading to a marked decline in intra-testicular hormone concentrations. Consistent with previous findings showing that obesity negatively affects LC survival by reducing the activity of key antioxidant enzymes and increasing the expression of proapoptotic markers [13], we demonstrated that HFD exposure impaired key antioxidant defenses—by reducing CAT, SOD2, and GPX1 levels—and, in turn, promoted LC apoptosis. Notably, this phenotype was accompanied by the concomitant upregulation of both circNFAT5 and NFAT5 protein. Their accumulation within LCs appears to drive cellular vulnerability under conditions of obesity, thereby establishing a mechanistic link between NFAT5 dysregulation and obesity-mediated LC dysfunctions.

In vitro investigations of TM3 cells, including RNA interference-mediated *NFAT5* knockdown and functional apoptosis assays, confirmed that NFAT5 serves as a central regulator of LC homeostasis. Mechanistically, our TM3 in vitro assays provided compelling evidence that NFAT5 orchestrates LC apoptotic fate. The NFAT5 silencing elicited a clear pro-survival shift. Conversely, chemically induced apoptosis drove a strong phosphorylation-dependent nuclear accumulation of NFAT5, which triggered the transcriptional activation of canonical proapoptotic genes. The robust NFAT5 nuclear shuttling, induced under apoptotic conditions, mirrors its canonical regulatory mechanism by which NFAT5 continuously cycles between the cytoplasm and nucleus through NLS/NES-dependent trafficking in response to cellular stress signaling [30,40]. Together, these complementary approaches demonstrate that NFAT5 nuclear shuttling critically governed the molecular program switch underling LC apoptosis. Several findings support the notion that NFAT2 plays a central role in regulating LC apoptosis [21,41], hence reinforcing a plausible NFAT family-dependent pathway driving LC survival under stress conditions. In particular, the characterized expression of NFAT2 in the mLTC-1 Leydig tumor cell line and its ability to perform nuclear shuttling, and, in turn, to enhance LC apoptosis, in an in vivo experimental model of stress [21,41] matched well with our data. In this light, the existence of a parallel NFAT2-mediated apoptotic axis in LC supports a broader model where multiple NFAT family members can integrate stress signals (hormonal, metabolic, osmotic) to modulate LC survival.

To determine whether paternal obesity imprints biological traits in offspring, we examined testicular features in male progeny derived from HFD mice (F1H). Interestingly, *circNFAT5* and NFAT5 expression, elevated in HFD fathers, recovered to physiological levels in F1H offspring, while oxidative stress, as well as LC apoptosis, was fully normalized. However, LC steroidogenesis remained incompletely restored: STAR, LHR, HSD3β, and CYP19A1 transcripts and proteins showed only a partial recovery, and intratesticular TT and E2 levels did not reach CTRL values. This dissociation between apoptosis recovery and steroidogenic deficiency suggests that different molecular pathways mediate LC demise *versus* LC functional maturation, thus highlighting the putative occurrence of NFAT5-dependent *versus* NFAT5-independent pathways. While NFAT5 recovery appears sufficient to restore LC survival and oxidative status, obesity-induced perturbations likely affect additional long-term steroidogenic regulatory mechanisms that persisted across generations. These findings align with reports showing that paternal obesity can transmit endocrine and metabolic vulnerabilities to offspring [31,32]. Our results extend this concept to testicular endocrinology, indicating that steroidogenic machinery may be particularly sensitive to paternal metabolic insults. However, while our data point toward the involvement of distinct regulatory pathways, the precise mechanisms underlying the long-lasting impairment of LC steroidogenesis remain to be fully elucidated. Characterizing these molecular underpinnings remains a pivotal challenge for future investigations. A major discovery of this study consists in the identification of epididymal NFAT5 deregulation as a putative novel mechanism potentially shaping sperm quality. We found that *cauda* epididymis from both HFD and F1H mice exhibited markedly increased NFAT5 expression accompanied by impaired sperm morphology, reduced sperm motility, altered sperm viability, and an increased frequency of acrosome-defective SPZ. Notably, this pattern was accomplished by a clear deregulation of 4HNE levels in both HFD and F1H SPZ, supporting the persistence of a spermatic oxidative stress signature—primarily epididymis-driven rather than directly testis-derived—potentially contributing to the defective post-testicular sperm maturation. In parallel, despite the enrichment of NFAT5 in epididymal tissue, its counterpart content in *cauda* SPZ was significantly reduced, suggesting a hampered proper epididymosome-mediated transfer of NFAT5 to maturing sperm cells. Interestingly, sperm cells are exquisitely sensitive to osmotic fluctuations within the epididymal lumen, a physiologically hyperosmotic niche required for their maturation [36,37,42]. Furthermore, the NFAT5 protein, by acting as master regulator of osmotic stress responses, normally aids cell adaptation to hypertonicity by promoting osmo-protective conditions [30,43]. In this light, our findings indicate that the adaptive epididymal upregulation of NFAT5 could act as a molecular sentinel not only of oxidative stress but also of an obesity-responsive osmotic stress state paternally transmitted across generations. Nevertheless, its defective transfer to sperm leads to a failure in the safeguard of proper epididymal maturation.

Notably, osmotic stress profoundly influences the molecular machinery governing cells’ epigenetic landscape in several biological systems. As a result, RNA output dynamically shifts during and after oxidative and osmotic perturbations [39,44]. Building on these findings, we further investigated whether the sperm circRNA landscape could be sensitive to paternal obesogenic insults and whether it might be functionally linked to the epididymal osmotic sentinel NFAT5. Here, we demonstrated that nine circRNAs previously identified as upregulated in HFD sperm [19] remained significantly elevated in sperm from F1H males. This epigenetic persistence occurred in parallel with increased FUS content, the main RBP modulating endogenous sperm backsplicing [45], in both HFD and F1H sperm. More intriguingly, FUS has emerged as an osmotic-sensitive RBP whose subcellular and functional dynamics are rapidly remodeled under hyperosmotic stress conditions [46,47]. These data clearly show that paternal obesity not only alters the spermatic circRNA profile but also amplifies this epigenetic insult in F1 offspring, as it is associated with deregulation of sperm morpho-functional parameters and the disruption of physiological oxidative balance. Hence, the epididymal overexpression of NFAT5 in F1H offspring, reported herein, was correlated with a marked deregulation of sperm oxidative and morpho-functional parameters, as well as of circRNA profile. Extending this paradigm to F2 generation and beyond would be necessary to distinguish between intergenerational and true transgenerational inheritance mechanisms. Collectively, these findings support a model in which epididymal NFAT5 dysregulation, combined with FUS-mediated backsplicing enhancement, creates a sperm oxidative environment predisposed to abnormal circRNA biogenesis. Whether these circRNAs directly modulate offspring health or reproductive outcomes remains an important question for future studies. In this context, a conceivable role for *circNFAT5* as molecular marker enabling the assessment of both testicular endocrine function and sperm epigenetic quality cannot be ruled out.

Although our findings strongly support a pivotal role for NFAT5 in regulating LC survival and sperm maturation, such evidence remains primarily correlative within the *in vivo* experimental framework. While our *NFAT5* in vitro silencing and apoptosis-induction assays demonstrate a functional involvement of NFAT5 in LC-programmed cell death, the absence of validation in a conditional knockout genetic model precludes the definitive establishment of a direct cause-and-effect relationship between NFAT5 dysregulation and the observed testicular and spermatic phenotypes. Addressing this limitation through targeted genetic models will be essential to fully delineate the specific contribution of NFAT5-dependent pathways to male reproductive dysfunction under metabolic stress.

## 5. Conclusions

In conclusion, our findings delineate a previously unappreciated double functional identity of NFAT5 in the male reproductive axis, acting as both a testicular executor and an epididymal sentinel of obesity-induced stress: While in the testis NFAT5 works as a cell-intrinsic regulator of oxidative stress and LC fate, in the epididymis, it assumes a complementary role as an osmotic stress sensor. Together, these dual actions position NFAT5 at the top of two hierarchical reproductive checkpoints: LC survival and epididymal sperm maturation. In this context, NFAT5 can be envisioned as a “metabolic time capsule”, encoding the history of paternal metabolic stress within the male reproductive tract and influencing the sperm epigenetic landscape in the next generations.

## Figures and Tables

**Table 1:** Primer sequence and annealing temperature.

Gene Primers	Sequences 5′-3′	Tm (°C)	Accession Number
***Lhr S*** ***Lhr AS***	GGGCTGGAGTCCATTCAGAC CACAGCAGTGGCTAGGGTAG	58	NM_013582.3
***Hsd3β S*** ***Hsd3β AS***	TGTGCATTAAGGCCCATGTTT TTGAGGGCCGTAATTATTGTGTT	56	NM_013821.3
***Star S*** ***Star AS***	GGCCACACATTTTGGGGAGA GGCGAACTCTATCTGGGTCTG	56	NM_011485.5
***Cyp19A1 S*** ***Cyp19A1 AS***	GCCCTTTCTTTATGAAAGCTC AGGCGTTAAAGTAACCCTGGA	58	NM_007810.4
***Bcl2 S*** ***Bcl2 AS***	CTTCTTTGAGTTCGGTGGGGT TCCACAAAGGCATCCCAGCCT	58	NM_009741.5
***Bax S*** ***Bax AS***	AGGATGCGTCCACCAAGAAGCT T CCGTGTCCACGTCAGCAATCA	58	NM_007527.4
***P53 S*** ***P53 AS***	CCTCAGCATCTTATCCGAGTGG T GGATGGTGGTACAGTCAGAGC	58	NM_011640.4
***NFAT5 S*** ***NFAT5 AS***	GTCACCACAGACCTCCCTGT GCGGGGAATAAAGAGGAGAC	60	NM_133957.3
***circNFAT5 S*** ***circNFAT5 AS***	AAAAGAGCACTCGTGCCAGA TCAGAGAATTGCATAAAATGGGG	56	mmu_circRNA_43429
***circMEMO1 S*** ***circMEMO1 AS***	ACTATGATGAATCCCAGGGGG CAGGGGCACATGATGGGAAG	56	mmu_circRNA_30887
***circDNAH S*** ***circDNAH AS***	TACACGGGCCCTGCATTGTA AGGAGAGACCCAGCATGTGTA	57	mmu_circRNA_20079
***circMAPT S*** ***circMAPT AS***	GTCAGGTCGAAGATTGGCTCT ATACTGGTTCAAAGCCTTGCC	56	mmu_circRNA_24229
***circDNER S*** ***circDNER AS***	TGTGTCCTAGACCCATGCAG TCTGCAACAAACTTCCAGACAC	56	mmu_circRNA_20427
***circCPSF6 S*** ***circCPSF6 AS***	TCGTTAGAAGATTTGCCCTTGT ACAACAGGACTCTGACCATGA	56	mmu_circRNA_22684
***circPTPN11 S*** ***circPTPN11 AS***	TACGGGGTCATGCGTGTTAG GGGGTGAAACCATTTGTCCG	56	mmu_circRNA_39252
***circADAM10 S*** ***circADAM10 AS***	CCTATGTCTTCACAGACCGGG TGGGGATAGTCTGAAGGTGC	56	mmu_circRNA_44583
***circRESP18 S*** ***circRESP18 S***	TCTCCCCAAAAGATGGTCAGG TGCCTTCGGGTACAATCTGG	56	mmu_circRNA_20362
***circTULP4 S*** ***circTULP4 AS***	ATAAACTTCAACCTGCGAGGC TCCCGGTTAATTCAGGAGCCA	56	mmu_circRNA_30196
***Cyclophilin-AS*** ***Cyclophilin-A AS***	TGGTCTTTGGGAAGGTGAAAG TGTCCACAGTCGGAAATGGT	56	NM_008907.2
***Rps18 S*** ***Rps18 AS***	GAGACTCTGGATGCTAACTAG GGACATCTAAGGGCATCACAG	56	NR_003278.3

## Data Availability

The datasets used during the current study are available from the corresponding author on reasonable request.

## Abbreviations

The following abbreviations are used in this manuscript:

LC	Leydig cells
CircRNA	Circular RNA
NFAT	Nuclear factor of activated T cell
FUS	Fused in sarcoma
HFD	High-fat diet
STAR	Steroidogenic Acute Regulatory protein
LHR	Luteinizing Hormone Receptor
HSD3β	3β-Hydroxysteroid Dehydrogenase
CYP19A1	Cytochrome P450 Family 19 Subfamily A Member 1
CAT	Catalase
SOD	Superoxide dismutase
GPX	Glutathione peroxidase
4HNE	4-hydroxy-2-nonenal
TT	Testosterone
E2	17-β-estradiol
PNA	Peanut Agglutinin
